# Personalized treatment of extensive stage small cell lung cancer: A case report and literature review

**DOI:** 10.3389/fonc.2022.956372

**Published:** 2022-08-11

**Authors:** Huaiyu Wang, Xuning Wang, Suxin Jiang, Jingna Zhu, Jie Liu, Chuanhong Zhou, Yanjun Zhu, Yong Han

**Affiliations:** Thoracic Surgery Department, Air Force Medical Center, Chinese People's Liberation Army (PLA), Beijing, China

**Keywords:** extensive-stage small cell lung cancer, anlotinib, durvalumab, long survival, NGS, bTMB

## Abstract

A 50-year-old female patient presented with post-exercise dyspnea in September 2016, and was subsequently diagnosed with SCLC with multiple brain and spinal metastases. The first-line treatment was etoposide combined with cisplatin and synchronously performed radiotherapy for the brain and spinal cord metastases. She was treated with anlotinib after disease progression in December 2018 and continued to have clinical benefit for nearly 25 months. Unexpectedly, the patient can still benefit from further combination treatment with durvalumab after another disease progression in February 2021. Thus, it may be a potential option to use anlotinib along with immunotherapy after the anlotinib resistance in SCLC, but more clinical data are still needed to confirm it. Moreover, ctDNA dynamic monitoring was performed and reflected the outcome of the process of treatment.

## Introduction

Small cell lung cancer (SCLC) is a malignant tumor with aggressive, rapid progression, and metastatic potential, accounting for about 10%–15% of lung cancer cases. At present, chemotherapy is still the main treatment for SCLC, and only a small number of patients can receive second-line treatment with limited benefit. Immune checkpoint inhibitors have shown good clinical effects in the first-line and backward treatment of SCLC, but their absolute benefit for SCLC is still limited (1, 2). Besides, there have been attempts to research antiangiogenic agents for SCLC, but previous studies have demonstrated that most antiangiogenic agents and the combination drug regimens for treating first-line or posterior SCLC have failed (3, 4). Thus, it is still necessary to explore more effective and safe new drugs and therapeutic schedules for SCLC. As a novel multi-target tyrosine kinase inhibitor (TKI), anlotinib can inhibit vascular endothelial growth factor receptor (VEGFR), fibroblast growth factor receptor (FGFR), platelet-derived growth factor receptor (PDGFR), and c-Kit at the same time, which can inhibit both angiogenesis and tumor growth. The study of ALTER1202 demonstrated that, compared with placebo, the progression-free survival (PFS) and overall survival (OS) were significantly improved in the third-line and above treatment of SCLC. Here, we present one case of an advanced SCLC patient who had received concurrent chemoradiotherapy (cCRT) and long-term benefit from anlotinib monotherapy after multiple lines of chemotherapy. Moreover, the combination of anlotinib and durvalumab still resulted in durable PFS and the tolerance was good enough after the disease progression.

## Case presentation

**Figure 1:**
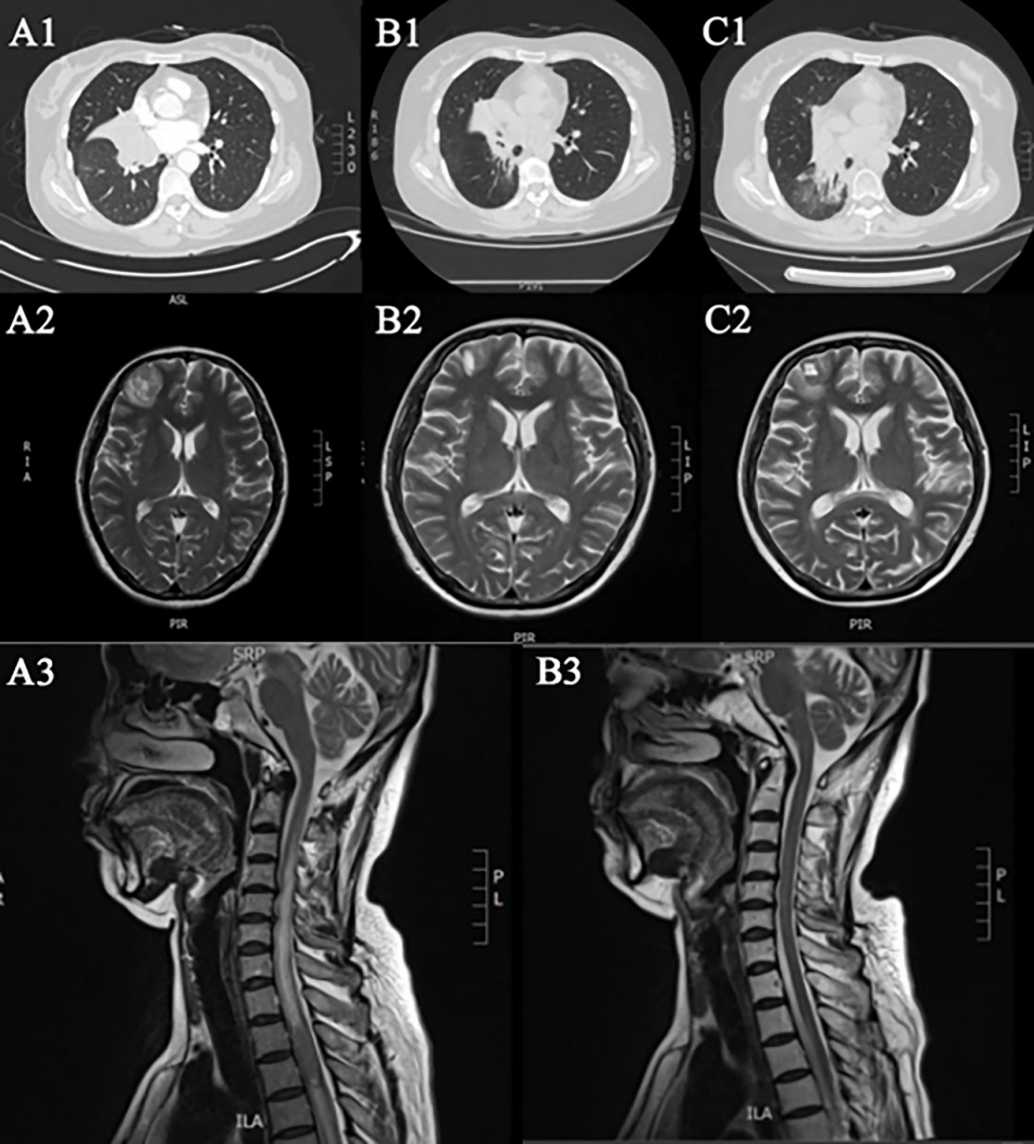
Imaging during chemotherapy. **Ai-iii** First diagnosis. **Bi-iii** After radiotherapy and chemotherapy. **(Ci, ii)** Progress after the second chemotherapy.

**Figure 2:**
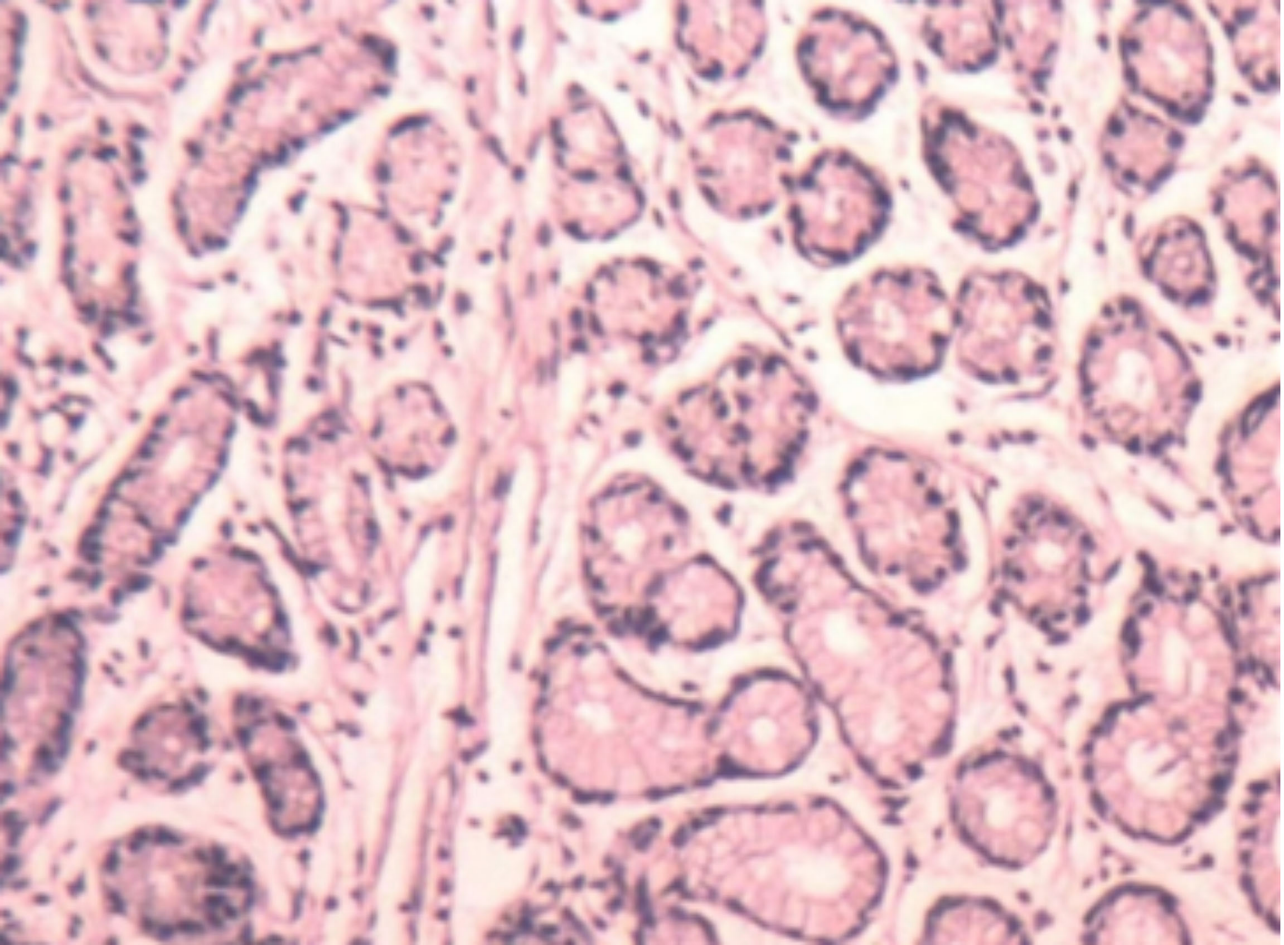
Bronchoscopic pathology showed small cell lung cancer.

A 50-year-old female was admitted to our hospital on 18 September 2016 due to post-exercise dyspnea and lower extremity parethesia. She had no cigarette history, no family history, but was allergic to sulfa. Enhanced chest CT indicated central lung cancer in the middle and lower hilum of the right lung with pulmonary atelectasis, invasion of the right hilar vessels, and mediastinal lymph node metastasis. Magnetic resonance imaging (MRI) of her brain and spinal cord revealed multiple brain and spinal metastases (**Figures 1Ai,ii**). Bronchoscopic biopsy pathology examination showed evidence of small cell carcinoma (**Figure 2**). An extensive stage of SCLC was diagnosed. The patient initially received inductive chemotherapy with “etoposide 100 mg/m^2^ (d1–d5) + cisplatin 120 mg/m^2^ (d1)” for two cycles on 23 September 2016 and 13 October 2016. Evaluation by CT scan showed a partial response (PR) based on the Response Evaluation Criteria in Solid Tumors (RECIST), version 1.1. Concurrent chemoradiotherapy (cCRT) was sequentially delivered, followed by two more cycles of adjuvant chemotherapy. The radiotherapy regimen including GTV/CTV 60/50 Gy/20 times for right lung and right hilar lymph node lesions, CTV 40 Gy/20 times for spinal cord metastasis at T2–3, CTV 30 Gy for intracranial metastasis of the whole brain (right frontal lobe, right paracentral lobule), and 50 Gy/15 times for GTV intracranial metastasis. During cCRT, the patient developed grade 1–2 gastrointestinal adverse reactions and grade 4 granulocytopenia and thrombocytopenia, so the adjuvant chemotherapy was suspended. The response was categorized as PR (**Figures 1Bi,ii**).

Unfortunately, the patient suffered multifocal metastases on October 9, 2018. PET/CT found a new lesion in the right lower lobe with multiple new hypermetabolic mediastinal lymph nodes. Meanwhile, a plasma ctDNA test was performed and six gene missense mutations were found, including CREBBP, KIT, MUTYH, MYC, PREX2, and SMO. Blood tumor mutational burden (bTMB), defined as the number of somatic, coding, base substitution, and indel mutations per megabase (Mb) of genome examined, was calculated from the GENETRON OncoPanscan 825 Panel NGS platforms. “TMB high” was defined as cases with a TMB of ≥10 muts/Mb, and the bTMB of this patient was 23.33 muts/Mb. Since there were no approved immunotherapy drugs for SCLC, the patient just received another two cycles of “etoposide + lobaplatin” chemotherapy on 18 October 2018 and 12 November 2018. But the response was categorized as progressive disease (PD) (**Figures 1Ci,ii**).

**Figure 3:**
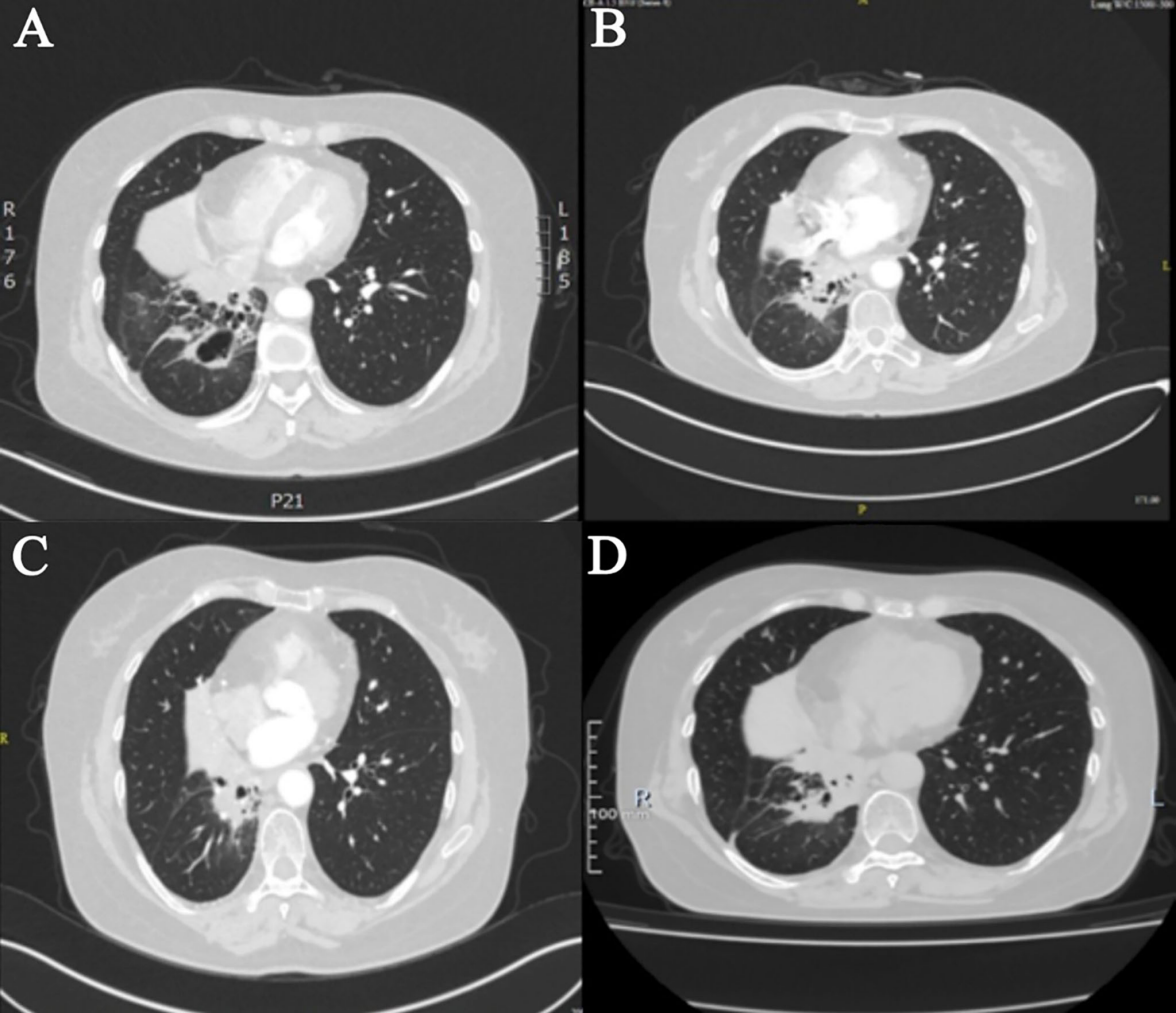
Imaging during Anlotinib treatment. **(A)** In December 2019, after anlotinib treatment, cavities formed. **(B)** In May 2020, void consolidation. **(C)** In November 2020, after the dose was increased, the lesions shrank again. **(D)** In October 2021, anlotinib combined with durvalumab shrink the lesion.

The patient then started taking anlotinib (12 mg d1–d14/q3w) from December 2018 until the scale of the pulmonary lesion shrank and cavitated (**Figure 3A**). Later, the dose of anlotinib was reduced to 10 mg because of paronychia. The consolidation of lung lesions was reviewed on 14 May 2020 (**Figure 3B**), and the dose of anlotinib was increased to 12 mg considering the risk of disease progression. During this period, the best efficacy was PR (**Figure 3C**), and no adverse reactions were reported.

In January 2021, she was referred for combination therapy of anlotinib along with durvalumab. As she had side effects during the previous anlotinib treatment, when adding durvalumab on this basis, to avoid the aggravation of side effects, the dose of durvalumab was adjusted and reduced to 1,000 mg q4w. The current clinical effectiveness was PR (**Figure 3D**). The last follow-up time was 28 March 2022. A ctDNA test was performed to monitor the effect of the treatment with bTMB reduced to 0 muts/Mb, which indicated the continuous benefit of anlotinib plus durvalumab.

**Figure 4:**
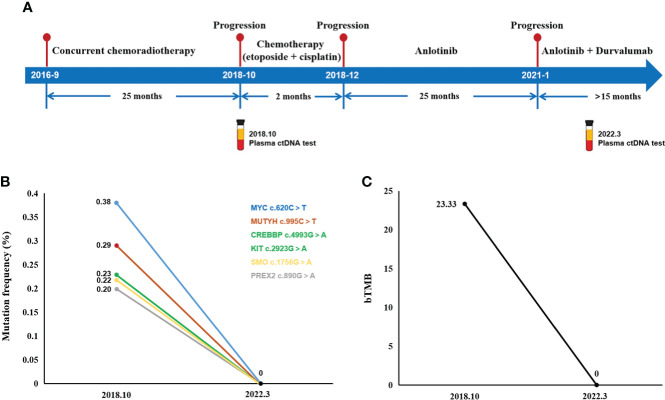
Treatment course and ctDNA NGS test results. **(A)** The complete treatment path of the patient. **(B)** Changes in gene mutation frequency and bTMB **(C)** between 2018 and 2022.

In summary, the complete treatment pathway for patients is demonstrated in **Figure 4**.

## Discussion

Extensive-stage SCLC accounts for about 60%–65% of SCLC (5). Previous studies have shown that the prognosis of extensive-stage SCLC is bleak, which has a median survival time of approximately 10 months and a less than 5% five-year survival rate with first-line chemotherapy regimens (6). Here, we report a case of a patient who achieved a response of nearly 25 months with the third-line treatment of anlotinib after the failure of second-line chemotherapy, and continued to achieve durable PFS and whose tolerance was satisfactory with subsequent anlotinib along with a durvalumab regimen after its progression.

Angiogenesis plays an important role in tumor growth, proliferation, and metastasis in SCLC (7). Thus, the anti-angiogenic drugs may play an important role in the treatment of SCLC as well. A study of 24 SCLC patients with the anti-angiogenic drug sunitinib, who had received at least one line of chemotherapy or concurrent chemoradiotherapy, showed that the ORR was 19%, and the median PFS and OS were 1.4 months and 5.6 months, respectively (8). Pazopanib is an inhibitor of the tyrosine kinase VEGFR2, PDGFR, and c-kit. In the study by Koinis et al. (9), patients after first-line platinum-based chemotherapy were included and divided into platinum-sensitive groups and platinum-resistant groups. In the overall 58 patients, the ORR was 13.8%, and the median PFS and OS were 2.5 months and 6.0 months, respectively. It seems that anti-angiogenic drug therapy plays a role in the second/third and above-line treatment of SCLC. But the number of study cases is small among those studies, which needs to be verified by a larger sample size. Anlotinib, as a novel TKI, can inhibit VEGFR, FGFR, PDGFR, and c-Kit at the same time, as well as inhibit angiogenesis and tumor growth. The ALTER1202 study, a randomized, double-blind, placebo-controlled multicenter phase II study of anlotinib in third-line and above treatment of SCLC, has been conducted in 2018. The results of ALTER1202 showed (10, 11) that patients with progressive or recurrent SCLC who were treated with anlotinib after second-line treatment had significant clinical benefits compared with placebo. Patients had a favorable clinical benefit of 3.4-month improvement in PFS (HR = 0.19, p <0.0001) and 2.4-month improvement in OS (HR = 0.53, p = 0.0029) in the anlotinib group. In our case, anlotinib was prescribed for the patient after disease progression from chemotherapy. In August 2019, anlotinib was approved for the treatment of SCLC that has progressed or relapsed after at least two prior chemotherapy regimens in China, and it is the only approved anti-angiogenic drug for the treatment of SCLC.

Immunotherapy has changed the treatment outcomes of advanced lung cancer. In the first-line treatment, atezolizumab combined with etoposide and carboplatin-improved OS from 10.3 months to 12.3 months compared with chemotherapy (1). The OS was also significantly better in the durvalumab + etoposide +/− carboplatin group than in the chemotherapy group (13.0 months *vs* 10.3 months) (12). Therefore, the above regimens have been approved for first-line treatment of extensive-stage SCLC in many countries. There are also case reports indicating that the PD-L1 antibody durvalumab can achieve a response of 7 months in third-line treatment of extensive-stage SCLC (13).

However, the clinical effectiveness of the anti-PD-1 inhibitors nivolumab and pembrolizumab in the third-line treatment of SCLC is still controversial. In the Checkmate032 study, the ORR was 10% in patients with relapsed SCLC treated with nivolumab 3 mg/kg, 23% in nivolumab (1 mg/kg) plus ipilimumab (3 mg/kg), and 19% in nivolumab (3 mg/kg) plus ipilimumab (1 mg/kg) (2). In this study, the duration of response was 17.9 months, the PFS was 1.4 months, and the OS was 5.6 months in the analysis of the third-line treatment with nivolumab alone (14). Similar results were shown in the KEYNOTE-028/158 study, which showed that the median PFS was 2.0 months and the median OS reached 7.7 months in the third-line and above treatment for SCLC (15). Based on the poor outcomes, Bristol-Myers Squibb (BMS) announced the withdrawal of nivolumab for the SCLC indication in the United States, and pembrolizumab also voluntarily withdrew its application for the SCLC indication in consultation with the FDA in 2021.

In recent years, the synergistic anti-tumor effect of anti-angiogenic drugs combined with immune checkpoint inhibitors has been supported by several studies. In preclinical (16) studies, anti-angiogenic drugs can promote the normalization of tumor vessels and regulate the immune microenvironment in many ways, which in turn activates the immune system. The mechanisms include promoting the maturation of dendritic cells, restoring the mobilization and infiltration of T cells, influencing the adhesion of lymphocytes, and reducing the induction and proliferation of inhibitory immune cells. At the same time, various innate and acquired immune cells are involved in the formation of blood vessels in tumors, and immune checkpoint inhibitors can promote tumor vascular normalization (17). This combination regimen has shown some efficacy for treating advanced SCLC. In the PASSION study of second-line treatment of SCLC (18), the efficacy results showed that the ORR was 33.9% and the PFS was 2.8 months in the overall population of apatinib combined with camrelizumab. The analysis showed that the ORR in the chemoresistant population and chemosensitive population was similar to that in the overall population.

However, although anti-angiogenic or anti-angiogenic drugs combined with immune checkpoint inhibitors for treating SCLC have shown preliminary efficacy, there are still many issues to be discussed, such as the suitable treatment population and the dose of treatment. In the combination therapy, the appropriate dose of anti-angiogenic drugs and the medication regimen are still worth exploring. Lin et al. (19) found that low-dose anti-angiogenic drug therapy may play an immune-promoting role by enhancing M1 polarization of macrophages and enhancing CD8+ T-cell function, while high-dose anti-angiogenic drug therapy may lead to immunosuppression of the microenvironment.

Moreover, liquid biopsy refers to the analysis of tumor-derived components in body fluids, among which circulating tumor DNA (ctDNA) has been used for dynamic monitoring of tumor changes, therapeutic effects, and patient prognosis in many cancers, including NSCLC (20), melanoma (21), and colorectal cancer (22). Compared with traditional tissue biopsy, ctDNA was noninvasive and could solve the problem of tumor heterogeneity. In SCLC, a few studies have shown that high pre-treatment ctDNA levels were associated with a poor prognosis in PFS and OS (23, 24), and plasma ctDNA could monitor dynamically the effect of treatment (25). The detection of ctDNA in LS-SCLC patients after curative treatment predicts disease recurrence and death (26). Additionally, ctDNA is a prognostic determinant in patients with SCLC treated with atezolizumab, and ctDNA is strongly associated with prognosis in SCLC patients treated with second-line immunotherapy (27). Although patients benefited from immunotherapy regardless of bTMB status in the IM133 clinical trial, it was found that patients with bTMB ≥16 were more likely to benefit from immunotherapy (28). In conclusion, liquid biopsy methods provide effective baseline analysis and longitudinal surveillance of LS and ES disease and have now been included in expanded SCLC studies and trials. We look forward to the results of these studies, particularly prospective studies of the role of ctDNA in predicting the efficacy of immunotherapy in SCLC.

Here, we found that patients with extensive SCLC can benefit from anti-angiogenic therapy plus immunotherapy depending on the situation. The efficacy may be assessed by ctDNA or bTMB, but further research is also needed.

## Data availability statement

The raw data supporting the conclusions of this article will be made available by the authors, without undue reservation.

## Ethics statement

Ethical review and approval was not required for the study on human participants in accordance with the local legislation and institutional requirements. The patients/participants provided their written informed consent to participate in this study. Written informed consent was obtained from the individual(s) for the publication of any potentially identifiable images or data included in this article.

## Author contributions

Authors HYW, XNW, SXJ, and JNZ collected the clinical information, diagnostic information, therapeutic information, and images of the patients. HYW wrote the manuscript. YH identified the case and submitted the manuscript. JNZ and CHZ revised the manuscript. YJZ and JL proofread the manuscript. All authors listed have made a substantial, direct, and intellectual contribution to the work and approved it for publication.

## Funding

This work was supported by the Wu Jieping Medical Foundation (No. 320.6750.2020-19-15).

## Acknowledgments

We are thankful to HY, KW, and YL for their kindness help in this case. All the authors are thankful to the patients who participated in this case.

## Conflict of interest

The authors declare that the research was conducted in the absence of any commercial or financial relationships that could be construed as a potential conflict of interest.

## Publisher’s note

All claims expressed in this article are solely those of the authors and do not necessarily represent those of their affiliated organizations, or those of the publisher, the editors and the reviewers. Any product that may be evaluated in this article, or claim that may be made by its manufacturer, is not guaranteed or endorsed by the publisher.
